# Characterization of the adaptive cellular and humoral immune responses to persistent colonization of *Brucella abortus* strain RB51 in a Jersey cow

**DOI:** 10.3389/fvets.2024.1367498

**Published:** 2024-07-26

**Authors:** Paola M. Boggiatto, Haley Sterle, Shollie Falkenberg, Kaitlyn Sarlo-Davila, Ellie J. Putz, Steven C. Olsen

**Affiliations:** ^1^Infectious Bacterial Diseases Research Unit, National Animal Disease Center, Ames, IA, United States; ^2^Immunobiology Interdepartmental Program, Iowa State University, Ames, IA, United States; ^3^Auburn University College of Veterinary Medicine, Auburn, AL, United States; ^4^Ruminant Diseases and Immunology Unit, National Animal Disease Center, Ames, IA, United States

**Keywords:** brucellosis, persistent shedding, RB51, *Brucella abortus*, adaptive immunity, Jersey cattle

## Abstract

*Brucella abortus* strain RB51 is the commercial cattle vaccine used in the United States (US) and many parts of the world against bovine brucellosis. RB51 was licensed for use in 1996, and it has been shown to be safe and efficacious in cattle, eliciting humoral and cellular responses in calves and adult animals. In 2017, an epidemiological trace-back investigation performed by the Centers for Disease Control and Prevention (CDC) identified human cases of brucellosis caused by infection with RB51. These infections resulted from the consumption of unpasteurized dairy products, which were traced back to otherwise healthy animals that were shedding RB51 in their milk. At the current time, six adult Jersey cows have been identified in the U.S. that are shedding RB51 in milk. One of the RB51 shedding cattle was obtained and housed at the National Animal Disease Center (NADC) for further study. Improved understanding of host cellular and humoral immune responses to RB51 in persistently colonized cattle may be achieved by the characterization of responses in shedding animals. We hypothesized, based on the lack of RB51 clearance, that the RB51 shedder animal has a diminished adaptive cellular immune response to RB51. Our data demonstrate that in the presence of persistent RB51 infection, there is a lack of peripheral anti-RB51 CD4^+^ T cell responses and a concurrently high anti-RB51 IgG humoral response. By understanding the mechanisms that result in RB51 persistence, the development of improved interventions or vaccinations for brucellosis may be facilitated, which would provide public health benefits, including reducing the risks associated with the consumption of non-pasteurized milk products.

## Introduction

In 2017, the CDC reported three cases of human brucellosis in the US attributed to *Brucella abortus* strain RB51 (1). All three cases were associated with the consumption of unpasteurized milk products (2). A traceback analysis identified two RB51 culture-positive cows from a farm tied to one of these cases, and whole-genome sequencing indicated a high degree of homology between the cow and human isolates (2). At the current time, six adult Jersey cows have been identified in the US that were shedding RB51 in milk with available records, suggesting that all animals were properly vaccinated between the ages of 4 and 12 months.

RB51 is a live attenuated strain of *B. abortus* and is the commercial vaccine for bovine brucellosis used in the US and many parts of the world. Most exposures to RB51 occur through occupational use, such as needle sticks when administering the vaccine. The 2017 reported human infections with RB51 were the first report of RB51 acquired through the consumption of raw milk in the US (1).

RB51 was licensed for use in 1996, and it is safe and efficacious in cattle. It elicits both humoral and cellular immune responses in vaccinated calves (3) and adult cows (4), and affords equal protection regardless of age group. RB51 was also shown to be safe and efficacious when administered at the maximally recommended dosage (3.2 × 10^10^ CFU) (5) or when multiple doses of RB51 are administered (4). Studies on the persistence of RB51 in cattle following experimental vaccination demonstrated that RB51 is cleared from the pre-scapular lymph node draining the site of inoculation within 12–14 weeks in most cattle (3, 5). RB51 has also been demonstrated to be safe for administration and efficacious in pregnant cattle when administered at 10^8^ and 10^9^ CFU doses at 6 months of gestation. Pregnant animals develop both cellular and humoral immune responses to vaccination, with no observations of abortions or placentitis due to RB51 vaccination (6).

In general, and based on data from experimental vaccination studies, cattle clear RB51 within 12 to 14 weeks post-vaccination (5) and do not shed the vaccine in nasal secretions, saliva, or urine. However, in some animals, vaccine clearance may be delayed and additional shedding of RB51 in milk or other secretions is possible (7). Immune mechanisms resulting in delayed clearance are not understood and warrant further exploration.

As mentioned above, RB51 vaccination results in cellular and humoral responses to the vaccine. Using a flow cytometry-based assay, we and others have previously shown that cellular immune responses to RB51 vaccination are characterized by proliferating and interferon-gamma (IFN-γ)-producing CD4^+^ T cells measurable within 8 weeks post-vaccination (8, 9). Additionally, anti-RB51 immunoglobulin G (IgG) responses can be measured as early as 2 weeks post-vaccination (5). Both cellular and humoral immune responses wane over time but can still be detected up to 24 weeks post-vaccination. Initial assessments of immune responses from RB51-shedding cattle indicated the absence of proliferative cellular responses and the presence of high antibody responses as compared to typical immune responses observed in RB51-vaccinated cattle (S. C. Olsen, National Animal Disease Center (NADC), Ames, IA, personal communication). Here, we sought to further characterize host immune responses to RB51 in an adult cow persistently colonized with the vaccine strain. We hypothesized, based on the lack of RB51 clearance and the above-mentioned preliminary data, that the RB51 shedder animal has a diminished adaptive cellular immune response to RB51.

In 2019, a Jersey cow from a hobby farm in Colorado was identified as an RB51 shedder. After preliminary testing at the National Veterinary Services Laboratory determined that RB51 was being shed in all four quarters of this cow, the animal was obtained by the NADC in Ames, Iowa for further research and observation to characterize peripheral immune responses elicited by persistent RB51 infection. Adaptive cellular and humoral responses to RB51 were analyzed prior to and following RB51 revaccination and compared to responses observed in non-Jersey cattle that were able to clear RB51. To our knowledge, the immune responses of RB51-shedding cattle have not been previously characterized.

## Materials and methods

### Animals, vaccination, and sample collection

An adult lactating Jersey cow (age estimated at 3–5 years) was obtained from a hobby farm in Colorado and housed outdoors at the NADC campus in Ames, Iowa. Hereford/Angus cross heifers (4–6 months old, *n* = 4) and Holstein/Angus cross heifers (18 months old, *n* = 8) were housed outdoors on the NADC campus in Ames, Iowa. Vaccinations with RB51 were performed via subcutaneous or intramuscular injection of 2 mL (10^10^ colony forming units (CFUs)) of the commercial *B. abortus* strain RB51 vaccine (Colorado Serum), per manufacturer’s recommendations. Non-vaccinated, control animals were left untreated. The Hereford/Angus cross heifers (*n* = 4) were also vaccinated with Bovi-shield Gold® 5 (Zoetis Animal Health, Kalamazoo, MI) containing bovine viral diarrhea virus (BVDV), per manufacturer’s recommendations. Blood samples were collected prior to and every 4 weeks post-vaccination via venipuncture of the jugular vein to assess cellular and humoral immune responses. Mammary gland secretions were collected from the mammary gland of the RB51 shedder cow to assess bacterial colonization. All studies involving animals were performed under the approval of the NADC Institutional Animal Care and Use Committee (IACUC).

### Enzyme-linked immunosorbent assay (ELISA)

Whole blood was collected via venipuncture of the jugular vein and placed into serum separator tubes. The blood samples were allowed to clot and then centrifuged at room temperature (RT) at 1,200 x *g* for 15 min. The serum was then collected, aliquoted, and frozen at −80°C until analysis. For the determination of humoral responses to RB51, ELISA for total IgG against RB51 antigen was performed as previously described (10). Briefly, 96-well flat bottom plates were coated with killed γ-irradiated RB51 antigen (10^8^ CFU/mL) in carbonate–bicarbonate buffer (0.18 M Carbinate-0.028 M sodium bicarbonate pH 9.6) overnight at 4° C and then blocked with SuperBlock™ blocking buffer (Thermo Fisher Scientific, Waltham, MA). Individual serum samples were diluted to 1:800, 1:1600, and 1:3200 and run in quadruplicates for each timepoint collected. For samples from the RB51 shedding cow, 1:2 serial dilutions up to 1:56000 were performed where indicated. The samples were incubated for 2 h at RT and washed 3 times with washing buffer (PBS containing 0.05% Tween 20). Horseradish peroxidase-conjugated anti-bovine IgG (Jackson ImmunoResearch, West Grove, PA) or anti-bovine immunoglobulin M (IgM) (Bethyl Fortis Life Sciences, Waltham, MA), diluted to 1:16000 and 1:25000, respectively, was added to each well and incubated for an additional 2 h at RT and again washed three times with washing buffer. The plates were developed using a 3,3′,5,5′-tetramethylbenzidine (TMB, Thermo Scientific) solution and stopped with 0.18 M sulfuric acid. The plates were analyzed on a microtiter plate and optical densities were read at 405 nm.

### *Brucella abortus* strain RB51 culture and PCR

Mammary gland secretions, from all four quarters, and blood samples were collected periodically from the RB51 shedding cow. The samples were plated onto tryptose agar plates containing 5% bovine serum as previously described (4, 11). Following incubation at 37^o^ C and 5% CO_2_ for 72 h, *Brucella* was identified based on colony morphology and growth characteristics and confirmed by a polymerase chain reaction procedure as previously described (12, 13).

### Peripheral blood mononuclear cell (PBMC) isolation and stimulation

Whole blood was collected via venipuncture of the jugular vein and placed into tubes containing 2x acid-citrate-dextrose (ACD) to prevent clotting. PBMCs were isolated via density centrifugation using Ficoll as described previously (9). PBMC viability and count were determined via the Guava Muse (Cytek Biosciences, Bethesda, MD) cell analyzer, and resuspended to a concentration of 1×10^7^ per ml. To determine proliferation and intracellular cytokine production, PBMCs were then stained with 1:10 CellTrace Violet (eBioscience, Thermo Fisher Scientific) solution, according to the manufacturer’s recommendations, and resuspended in complete RPMI media. PBMCs were then plated, 1×10^6^ per well, in 96-flat bottom plates, and were left unstimulated, stimulated with killed RB51 antigen, or stimulated with Concanavalin A (ConA; 0.5 μg/well; Sigma). The plates were placed in an incubator for 7 days at 37° C with 5% CO_2_. Approximately 16 h prior to harvest, all wells were treated with a 1x GolgiStop solution (eBioscience, Thermo Fisher Scientific) or a 1x Cell Stimulation Cocktail plus GolgiStop solution (eBioscience, Thermo Fisher Scientific), according to the manufacturer’s recommendation.

To assess inhibitory receptor mRNA expression, PBMCs were isolated as described above and 1×10^6^ PBMCs were plated in duplicates onto 96-flat bottom plates. PBMCs were then left unstimulated, stimulated with killed RB51 antigen, or stimulated with BVDV and incubated at 37° C with 5% CO_2_ overnight.

### Flow cytometry and PrimeFlow

At the end of the incubation period, cells were prepared for flow cytometry staining as described previously (9). Briefly, PMBCs were transferred onto round-bottom 96-well plates, centrifuged at 300x *g* at RT, and then washed in 1x Dulbecco’s phosphate-buffered saline (DPBS) twice. PMBC were then incubated with a fixable viability dye (Invitrogen, Thermo Fisher Scientific) according to manufacturer’s recommendations, and washed once in DPBS and once in FACS buffer (0.5% fetal bovine serum (FBS) in PBS). PBMCs were then resuspended with gentle vortexing, and incubated with antibodies against bovine CD4 (FITC-labeled; CC30) (Bio-Rad Antibodies, Hercules, CA) for 15 min at RT, followed by two additional washes in FACS buffer. For intracellular staining, PBMCs were fixed and permeabilized using a permeabilization kit according to the manufacturer’s recommendations (BD Biosciences, San Jose, CA). Intracellular cytokine staining was performed using an anti-bovine IFN-γ antibody (PE-labeled; CC302). PBMCs were then washed twice in wash buffer (BD Biosciences), once in FACS, and then resuspended in FACS buffer until analysis. The data were collected using the BD Symphony flow cytometer (BD Biosciences) using the DIVA software and analyzed using FlowJo® (FlowJo, Ashland, OR).

For the analysis of inhibitory marker gene expression, PBMCs were left unstimulated or stimulated with killed RB51 antigen as indicated above, or with the previously described (14) BVDV-2 strain (PI-28) at an approximate multiplicity of infection (MOI) of 1 for approximately 24 h. PBMCs were washed in DPBS, surface stained with anti-bovine CD4 antibody as described above, and prepared for PrimeFlow as described in (15). Detection of bovine programmed-cell death 1 (PD-1) and lymphocyte activating gene 3 (LAG-3) mRNA expression was accomplished by gene-specific oligonucleotide (mRNA) target probes designed and manufactured by Thermo Fisher Scientific and are considered proprietary by the company. The data were collected using the BD Symphony flow cytometer (BD Biosciences) using DIVA software. The data were analyzed using FlowJo® (FlowJo). The gating strategy used for flow cytometry analysis is shown in Supplementary Figure 1.

## Results

### Bacterial colonization

Upon arrival at the NADC campus in February 2021, secretions from the mammary gland and blood samples were collected and plated to determine the presence of RB51. The mammary gland secretions were positive for RB51 growth at the time of arrival, while the blood samples collected were negative for the presence of RB51 (Table 1). Identification of RB51 growth was made through morphology and was confirmed as *Brucella* spp. via PCR. Additionally, mammary gland secretions were subsequently collected at various time points over 34 months and assessed for RB51 growth. At all time points analyzed, RB51 growth was recovered (Table 2). The blood samples remained negative for the presence of RB51.

**Table 1:** Assessment for the presence of *Brucella* spp. via culture and PCR of mammary gland secretions and blood samples from the RB51 shedder cow.

Sample	Culture	PCR (ΔCt value)
Blood	Neg	35.3
Milk	Pos	19.93

**Table 2:** Assessment for the presence of *Brucella* spp. in mammary gland secretion samples from the RB51 shedder cow via culture.

Dates tested	Milk culture
2/11/21	Pos
6/29/21	Pos
6/29/23	Pos
12/14/23	Pos

### Initial serological and cellular responses to RB51

**Figure 1:**
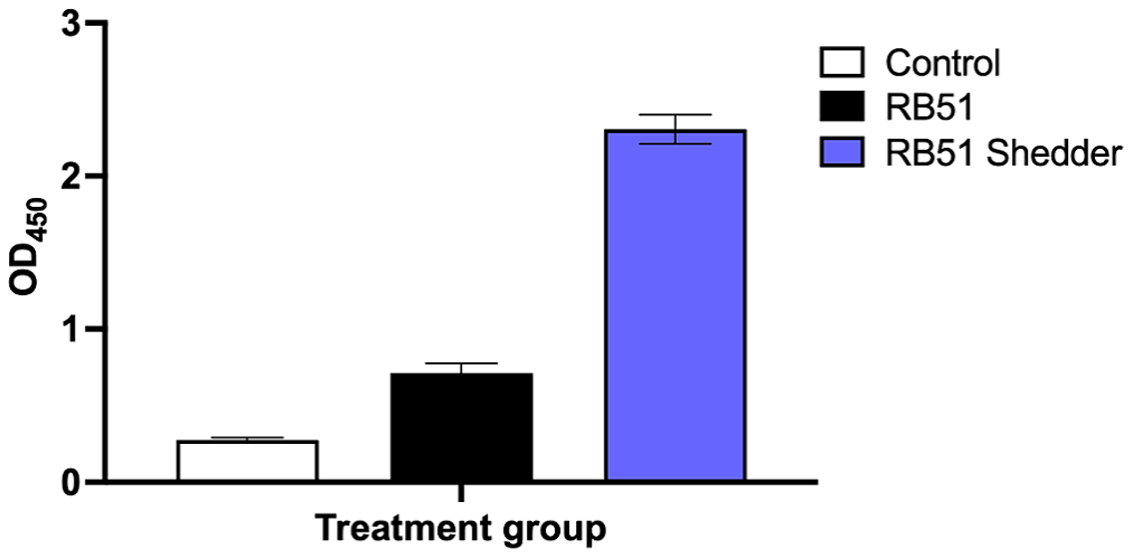
Anti-RB51 serological responses following RB51 vaccination. Shown are mean ELISA OD values for anti-RB51 IgG from serum samples collected from the RB51 shedder cow (blue), cattle vaccinated with RB51 at 8-week post-RB51 vaccination (black), and non-vaccinated controls (white). Shown are mean OD values and error bars indicate ± SEM.

Humoral responses, specifically anti-RB51 IgG, were assessed via ELISA from serum samples collected from the RB51 shedder cow and compared to cattle with known RB51 vaccination status. Initial assessment showed that the RB51 shedder cow had OD values at least 2x that of RB51 vaccinated cattle, even at the peak of IgG antibody responses at approximately 8 weeks post-vaccination (Figure 1). These data are consistent with previous reports from other RB51 shedder Jersey cows, where antibody responses to RB51 are high as compared to other vaccinated cattle (Olsen, unpublished results).

**Figure 2:**
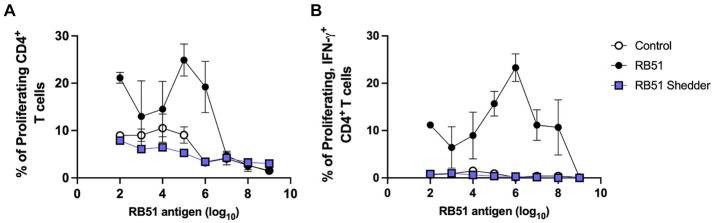
Lack of CD4^+^ T cell proliferative and IFN-γ responses in the RB51 shedder cow. PBMC from the RB51 shedder cow (blue), RB51 vaccinated cattle (black), and control animals (white) were isolated and stimulated with varying concentrations of the killed RB51 antigen. Cells were then analyzed for proliferation **(A)** and proliferative and IFN-γ **(B)** responses. Shown are mean frequencies ± SEM.

We also characterized the peripheral cellular immune response to RB51 and compared it to that of RB51-vaccinated cattle. Using an *in vitro* recall response assay, we *in vitro* stimulated PBMC with different concentrations of killed RB51 antigen and assessed CD4^+^ T cell proliferation and IFN-γ responses (Figure 2). As expected, cattle vaccinated with RB51 (Hereford/Angus cross, *n* = 4) show a dose-dependent response to increasing amounts of RB51 antigen both in the frequency of proliferating (Figure 2A) and proliferating and IFN-γ-producing CD4^+^ T cells (Figure 2B). In contrast, the RB51 shedder cow did not respond to RB51 regardless of the amount of antigen provided either via proliferation and/or IFN-γ production (Figures 2A,B). The cellular responses of the RB51 shedder cow are very similar to those of non-vaccinated controls.

### Humoral and cellular responses post-RB51 vaccination

In order to further characterize the immune response to RB51 antigen in the RB51 shedder cow, we opted to re-vaccinate the shedder cow with RB51 and track humoral and cellular responses at various time points post-vaccination. Concurrently, we vaccinated naïve cattle (Holstein/Angus crosses, *n* = 8) with RB51 and followed those responses for comparison.

**Figure 3:**
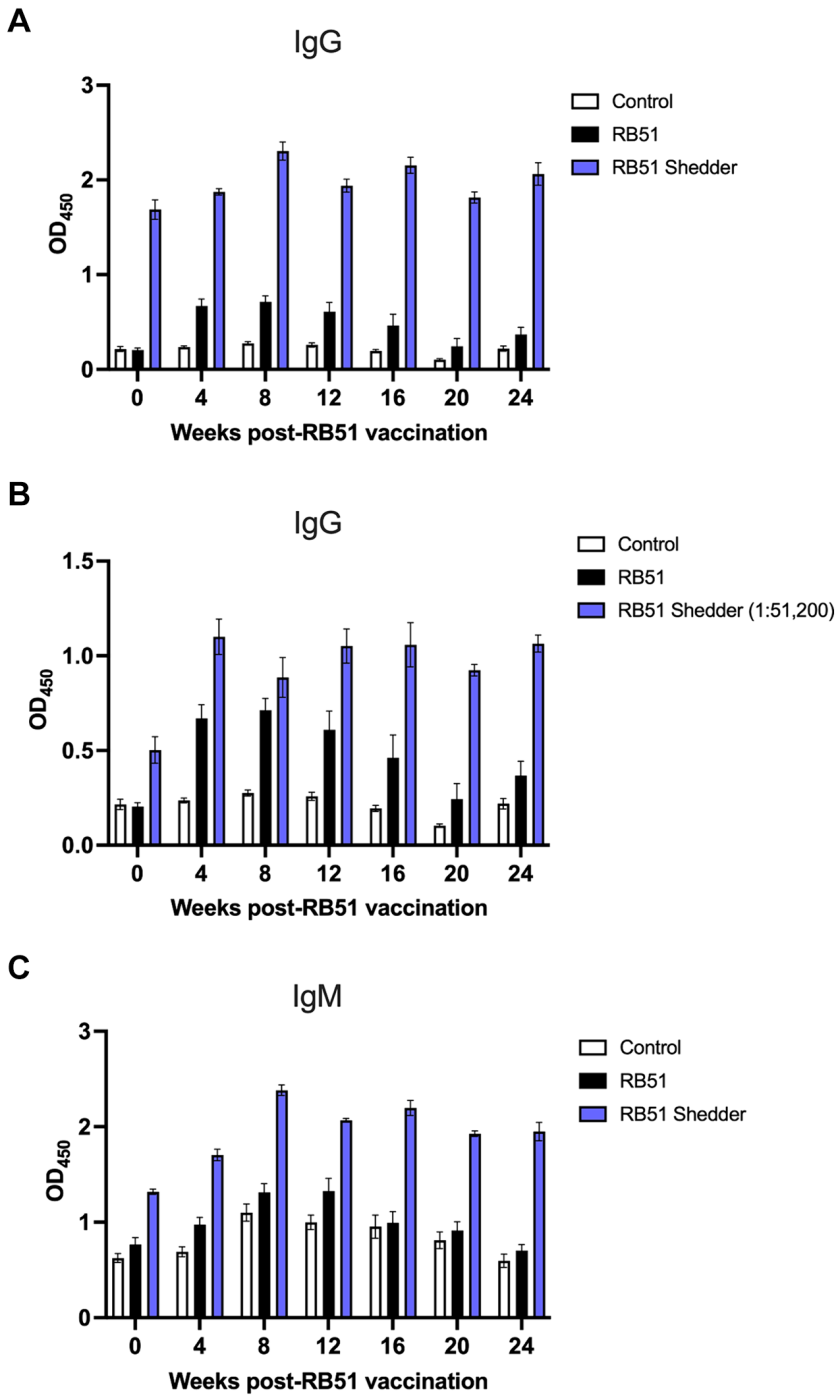
Comparison of serological responses to RB51 vaccination between conventional cattle and the RB51 shedder cow. Serum samples were collected prior to and at 4-week intervals following vaccination with RB51. Shown are IgG and IgM responses against RB51 antigen via ELISA in non-vaccinated animals (white), RB51 vaccinated animals (black), and the RB51 shedder cow upon revaccination with RB51 (blue). The results for ant-RB51 IgG are presented with all serum samples diluted 1:1600 **(A)** and also with the RB51 shedder cow serum samples diluted 1:51,200 **(B)**. The results for anti-RB51 IgM responses are shown at 1:1600 dilution **(C)**. Shown are mean OD values, and error bars indicate ± SEM.

As expected, in conventional non-shedder cattle, RB51 vaccination results in the induction of an IgG humoral response by 4 weeks post-vaccination and begins to wane at approximately 16 weeks post-vaccination (Figure 3A). At the time of vaccination, OD values for anti-RB51 IgG antibody in the RB51 shedder were already higher as compared to all other cattle. Following RB51 vaccination, we observed a slight increase in OD values, which were sustained through all the time points analyzed (Figure 3A).

To fully appreciate the extent of the RB51 shedder cow’s humoral response to RB51, we performed serial dilutions of the serum at all time points shown. Figure 3B depicts the RB51 shedder’s anti-RB51 IgG OD values at a 1:51,200 dilution as compared to control and RB51 vaccinated conventional cattle serum diluted at 1:1,600. At this high dilution, it is possible to appreciate the increase in anti-RB51 IgG OD values for the RB51 shedder cow following RB51 revaccination. Furthermore, it highlights the magnitude of this response as compared to other cattle vaccinated with RB51.

Additionally, we measured the anti-IgM response to RB51 following RB51 vaccination. At the time points analyzed, we did not observe a significant increase in anti-RB51 IgM OD values in conventional cattle as compared to control animals (Figure 3C). Interestingly, however, compared to both control and vaccinated cattle, the RB51 shedder cow had higher OD values of IgM pre-vaccination, and following vaccination, those levels increased, peaking at approximately 8 weeks post-vaccination, and remained high at all time points analyzed (Figure 3C).

**Figure 4:**
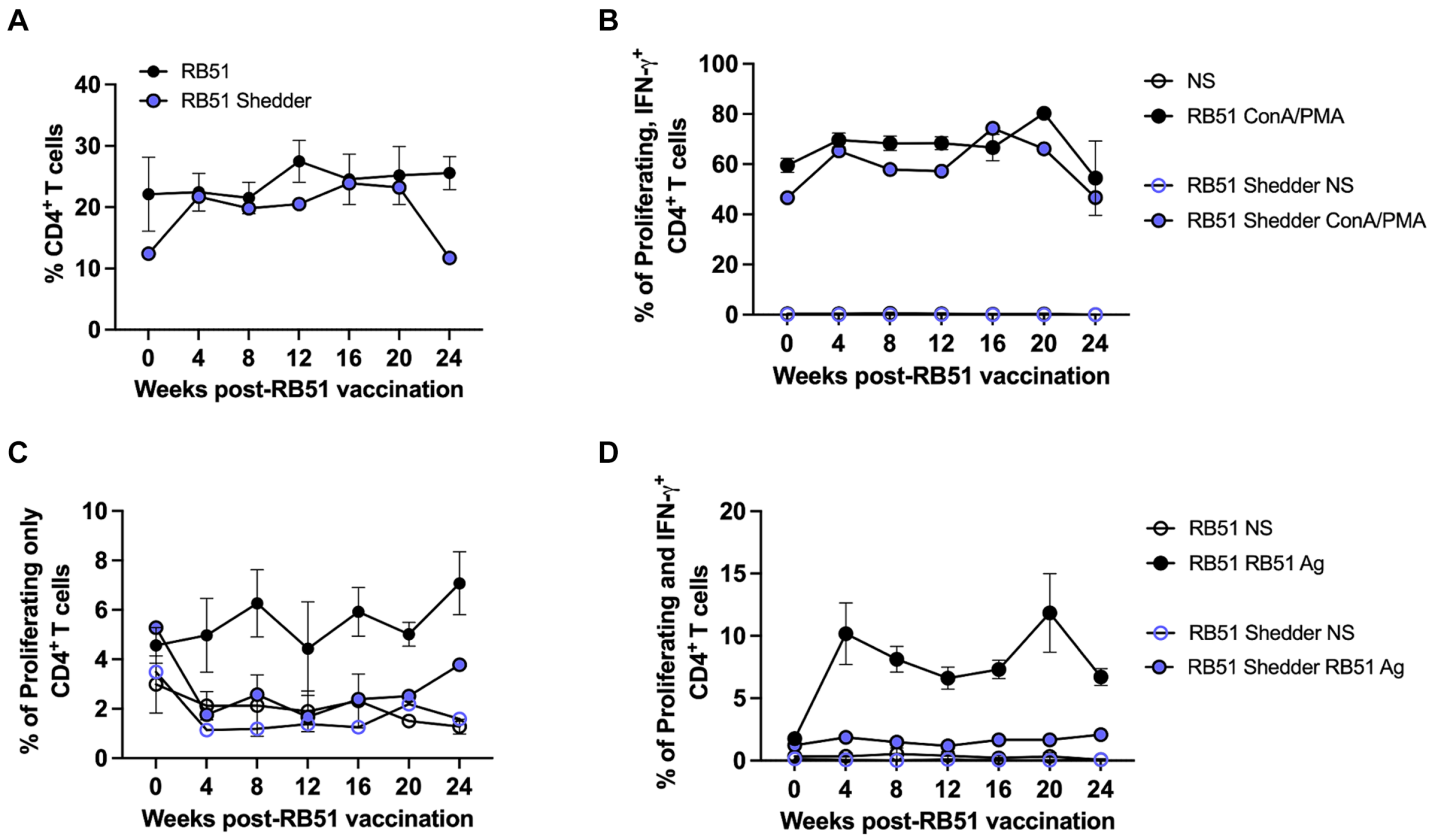
RB51 shedder cow fails to respond to revaccination with RB51. PBCM were isolated from whole blood samples collected prior to and at 4-week intervals post-vaccination with RB51. Shown are the frequency of circulating CD4^+^ T cells **(A)** and a comparison of proliferation and IFN-γ production by CD4^+^ T cells following stimulation with pan-T cell stimulators **(B)**. PBMC were also stimulated with killed RB51 antigen (RB51 Ag) or left unstimulated (NS) to assess RB51-specific CD4^+^ T cell proliferation **(C)** and proliferation and IFN-γ production **(D)** from the RB51 shedder cow and conventional cattle. Shown are mean frequencies ± SEM.

We also assessed cellular responses at various time points after RB51 vaccination. The frequency of CD4^+^ T cells between the RB51 shedder and conventional cattle were similar at all time points analyzed (Figure 4A), suggesting that the lack of responses was not due to a decreased CD4^+^ T cell subset. We then sought to determine if PBMCs from the RB51 shedder were capable of proliferating and producing IFN-γ by stimulating cells using pan-T cell stimulators of proliferation and cytokine production, Concanavalin A (ConA) and PMA/Ionomycin, respectively. As shown in Figure 4B, CD4^+^ T cells from the RB51 shedder are capable of both proliferation and IFN-γ production, similar to frequencies of response observed in conventional cattle. These data suggest that RB51 shedder PBMC are capable of responding to stimulation and that the previously observed lack of a response is not due to a pan-inhibition of T cells. We then assessed responses to RB51 at various time points post-vaccination. When PBMCs from conventional RB51-vaccinated cattle are stimulated *in vitro* with RB51 antigen, we observe an increase in the frequency of proliferating (Figure 4C) and proliferating and IFN-γ-producing (Figure 4D) CD4^+^ T cells. In contrast, we did not observe any RB51-specific CD4^+^ T cell proliferative and/or cytokine responses from the RB51 shedder at any of the time points analyzed (Figures 4C,D).

### Expression of inhibitory receptors

**Figure 5:**
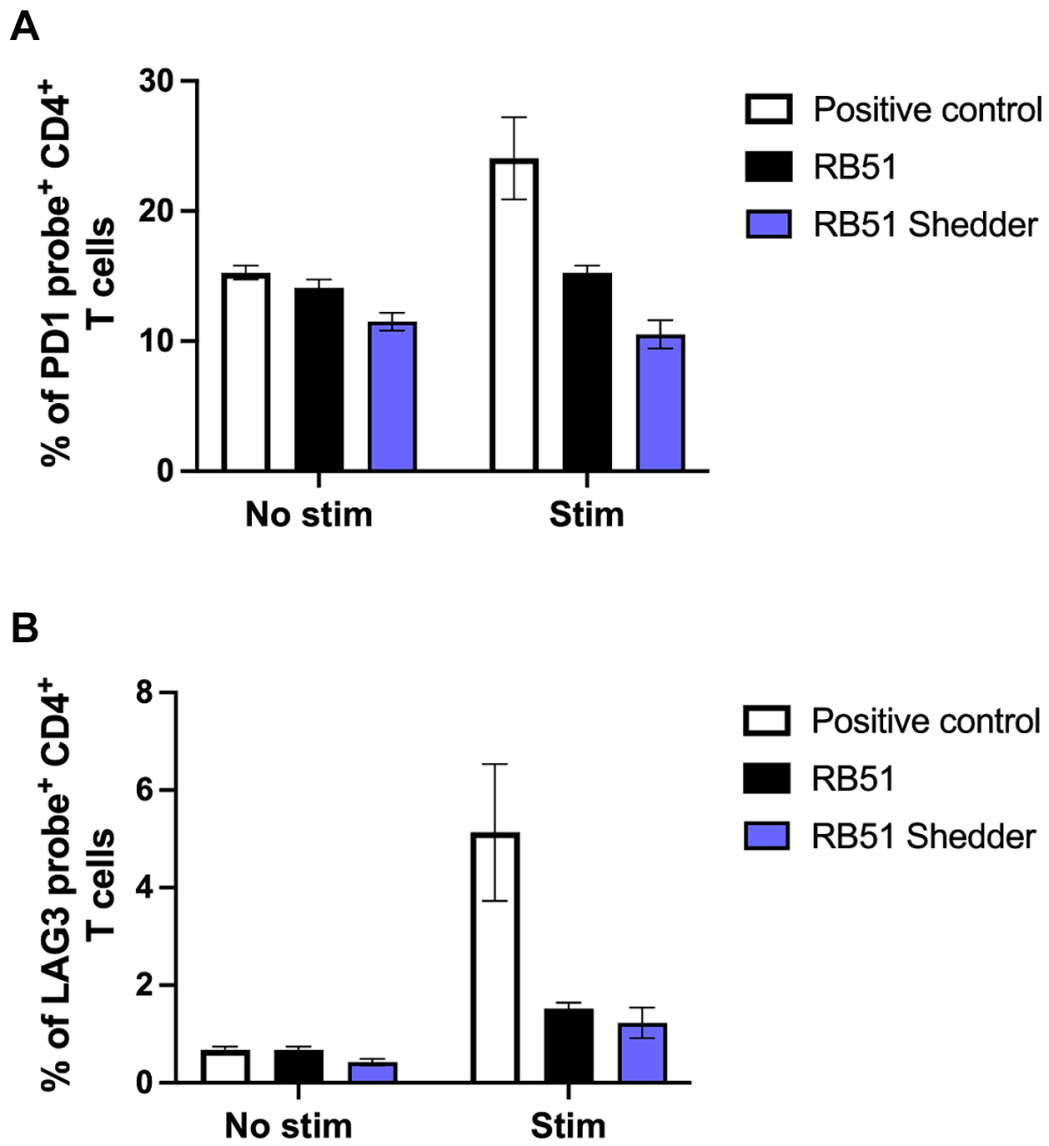
PD-1 and LAG-3 mRNA expression on CD4^+^ T cells are not increased following RB51 antigen stimulation. PBMC from conventional cattle (black) and the RB51 shedder cow (purple) were assessed for expression of exhaustion markers PD-1 **(A)** and LAG-3 **(B)** mRNA following *in vitro* RB51 antigen stimulation. PBMC were either left unstimulated (no stim) or stimulated with either RB51 antigen (black). BVDV stimulation of PBMC from BVDV-vaccinated cattle stimulated with BVDV (white) was used as a positive control for PD-1 and LAG-3 mRNA upregulation. Shown is the mean frequency of positive cells for either marker, ± SEM.

To determine the mechanism of action driving the lack of CD4^+^ T cell responsiveness in the RB51 shedder cow, we explored whether exhaustion of T cells was playing a role. Therefore, we assessed the frequency of CD4^+^ T cells expressing one of two known exhaustion markers via PrimeFlow: programmed cell death protein 1 (PD-1) (16) and lymphocyte activation gene 3 (LAG-3). As a positive control for inhibitory receptor expression, we utilized BVDV *in vitro* stimulation (17) of BVDV-vaccinated cattle. When PBMCs from conventional cattle vaccinated with BVDV are stimulated with BVDV, we observe an increase in the frequency of CD4^+^ T cells expressing PD-1 (Figure 5A) and LAG-3 (Figure 5B) mRNA as compared to non-stimulated PBMCs. We then tested whether stimulation with RB51 antigen would induce PD-1 or LAG-3 expression on PBMC from either the RB51 shedder cow and/or conventional cattle. We did not observe an increase in the frequency of CD4^+^ T cells expressing mRNA for either marker in PBMC from conventional cattle vaccinated with RB51 or PBMC from the RB51 shedder cow when compared to non-stimulated cells (Figures 5A,B). These data would suggest that T cell exhaustion may not be the mechanism for a lack of CD4^+^ T cell responses to RB51 antigens in RB51 shedder cows.

## Discussion

Vaccination of cattle with RB51 has commonly been practiced in the US since commercial licensure in 1996. The vaccine has been demonstrated to be safe when administered to calves, adult cattle, and even pregnant cows (3, 5, 6). However, it was also shown to persist in some cattle following experimental infection (5), although the incidence and significance of such persistence remain unknown. Since 2017, a total of seven animals, all Jersey adult cows, have been identified as persistent RB51 shedders. The identification of some animals resulted from epidemiological traceback investigations by the CDC due to human cases of brucellosis caused by RB51 infection (2), whereas others did not involve human infection. At the current time, the true incidence of persistent RB51 infection in cattle remains unknown.

The mechanisms resulting in RB51 persistence in some cattle remain poorly understood. Interestingly, all cattle identified to date are Jerseys, suggesting that there may be a genetic component driving long-term RB51 persistence. In food-producing animals, it is well recognized that breed-dependent differences in genetic variability influence disease resistance vs. susceptibility (18). Within dairy breeds, Jersey and Holstein cattle are the most common breeds and comprise most dairy herds in the US. Dairy cattle breeds have undergone extensive genetic manipulation in order to increase milk production yields or quality (19), at times resulting in decreased fertility and health (20). However, no conclusive data demonstrate an increased susceptibility of Jerseys to brucellosis.

In general, RB51 vaccination results in the induction of both cellular and humoral responses. The cellular immune response is a T helper 1 (T_H_1), characterized by CD4^+^ T cells that proliferate and produce IFN-γ in response to *in vitro* antigen stimulation (6, 8, 9). Induction of a T_H_1 response is necessary to clear intracellular bacterial pathogens such as *Brucella* spp. However, when peripheral cellular immune responses were measured in the RB51 shedder cow, we did not observe any measurable proliferative or IFN-γ responses as compared to other vaccinated cattle, following *in vitro* stimulation with various concentrations of RB51 antigen (Figure 2). The frequency of CD4^+^ T cells in circulation was similar between conventional cattle and the cow persistently infected with RB51, suggesting that the lack of a response is not due to a reduced CD4^+^ T population. Additionally, when PBMCs from the RB51 shedder cow are stimulated with pan-T cell stimulators, these cells proliferate and produce IFN-γ at similar frequencies as conventional cattle (Figure 3). Altogether, these data indicate that CD4^+^ T cells from this animal are capable of proliferation and cytokine production, suggesting that there is no intrinsic defect in their ability to respond and that the lack of responses is specific to the RB51 antigen.

We hypothesized that the lack of measurable peripheral responses may be due to the timing of the last vaccination and that sufficient time had transpired, resulting in a very low level of memory T cells whose response was below the level of detection of our assay. In order to boost the cellular immune response in the RB51 shedder cow, we revaccinated with RB51 and measured cellular and humoral responses over a period of 24 weeks. Similar to our initial findings, we were not able to detect proliferative or IFN-γ CD4^+^ T cell responses in the RB51 shedder cow at any of the timepoints analyzed (Figure 4). Altogether, these data indicate that a peripheral anamnestic cellular response is not present and suggest a lack of RB51-specific memory T cell trafficking in the peripheral blood of the RB51 shedder cow.

The absence of a measurable peripheral CD4^+^ T cell response to RB51 could be explained via several mechanisms. The simplest of which is that a T cell response does exist, but it is primarily found outside of the peripheral blood. At this time, we have not assessed the presence of RB51-specific T cells in other tissues, such as lymph nodes. Sequestration of antigen-specific cells away from the periphery is possible and may explain the lack of measurable responses in the blood. The mammary gland and supramammary lymph nodes are preferred sites for *B. abortus*, and we have recovered high bacterial loads from the RB51 shedder cow (Table 1). After infection or vaccination, memory T cells typically relocate within specific lymphoid and non-lymphoid tissues, based on their effector phenotype and initial sites of antigen encounter and priming (21). This relocalization of memory T cells within specific sites allows for a more effective and rapid response upon antigen re-encounter. For the RB51 shedder cow, the lymph nodes, particularly those lymph nodes associated with the mammary gland, or within the mammary gland itself, would be possible sites of memory T cell localization. Currently, we have not evaluated cells localized within lymph nodes, using lymphadenectomy or lymph node biopsies, to determine if RB51-specific T cells are present.

Alternative explanations may be related to processes that directly affect T cells and their function. For example, peripheral tolerance to foreign antigens (i.e., non-self) can occur through a variety of immune mechanisms, including ignorance, energy, and exhaustion (22). Ignorance results from very low levels of or no antigen being presented to cognate naïve T cells. Anergic T cells are naïve T cells that have seen sufficient levels of cognate antigen to become stimulated but lack the appropriate co-stimulatory signals. In contrast, exhaustion occurs when activated, functional T cells are exposed to high levels of persistent antigen over time (23) and progressively lose their functional phenotype. This phenomenon has been observed and extensively studied in various infectious (24) and non-infectious disease models (25). Given the high levels of anti-RB51 IgG antibodies produced, we suspect that at some point the RB51 shedder cow did develop an RB51-specific CD4^+^ T cell response that allowed for an isotype switch, suggesting that ignorance and energy are not at play in this system.

T cell exhaustion is a progressive and stepwise loss of effector function that can be characterized by changes in phenotypic, functional, gene expression, metabolic, and epigenetic attributes (26–28). These changes include diminished proliferation and differentiation, loss of cytokine production such as interleukin (IL)-2, tumor necrosis factor (TNF)-α, and IFN-γ, and increased expression of inhibitory surface receptors such as PD-1 and LAG-3. Immune exhaustion in cattle has been described previously, demonstrating similar losses of effector functions, as observed in murine models, in bovine leukemia virus (BLV) infection, Johne’s disease, and bovine anaplasmosis (29). In order to determine if exhaustion is playing a role during persistent RB51 colonization, we assessed the expression levels of PD-1 and LAG-3 mRNA in PBMC from the RB51 shedder cow and conventional cattle following *in vitro* stimulation with RB51 antigen. We did not observe an increase in the expression of either of these two markers in CD4^+^ T cells from the RB51 shedder cow as compared to other vaccinated cattle. This initial assessment would suggest that exhaustion may not be driving the observed T-cell dysfunction in this system. However, it should be noted that, despite the lack of PD-1 or LAG-3 mRNA expression detected, the last stage in the process of exhaustion is the deletion of exhausted T cells (27). As such, it is possible that at the time of our analysis, most (if not all) RB51-specific CD4^+^ T cells could have been deleted due to the persistently high antigen load in this animal.

Dysfunction of antigen-specific CD4^+^ T cells, in the context of a chronic high antigen load, may also be caused by other mechanisms. For example, the presence of regulatory T cells (Treg) has been shown to dampen T cell responses following persistent bacterial, viral, and parasitic infections (30, 31). A Treg response acts to control inflammation and tissue injury in the face of continued T cell responses and inflammation. Additionally, other mechanisms may include antigen-specific T cell clone deletion, as demonstrated in a mouse model of malaria, where adoptively transferred CD4^+^ T cells are deleted from the periphery and tissues in an IFN-γ-dependent manner following infection (32). Similarly, in mouse models of Bacillus Calmette–Guerin (BCG) infection, IFN-γ-mediated depletion of antigen-specific CD4^+^ T cells has also been characterized (33, 34), demonstrating a possible role of continued IFN-γ responses as a mechanism for infection-mediated T-cell apoptosis. However, the mechanism resulting in a lack of RB51-specific CD4^+^ T cell responses in the context of persistent RB51 remains unknown.

Despite the lack of measurable RB51-specific CD4^+^ T cell responses, we do observe high OD values for anti-RB51 IgG in the RB51 shedder cow. Classically, class-switched IgG responses are characterized by the presence of T-cell help (35). Therefore, the presence of IgG antibodies against RB51 in the shedder cow would suggest that at some point following RB51 vaccination, this animal developed RB51-specific CD4^+^ T cells. As mentioned above, we cannot discard the possibility that there is a CD4^+^ T cell response present outside of the peripheral blood, providing help for this IgG response. Yet, humoral responses to T cell-independent (TI) antigens are possible, which are typically directed at non-proteinaceous antigens with highly repetitive motifs, such as bacterial polysaccharides, and often result in IgM-driven, short-lived responses. While TI IgG responses have been characterized and can provide protection from infection, these are not classically characterized by high-affinity IgG, nor are they typically long-lasting responses (36, 37). However, long-lived protective IgG responses in the absence of CD4^+^ T cells were demonstrated in a mouse model of polyomavirus infection (38). This long-lasting IgG response was dependent on the presence of inflammatory signals and a high persistent viral load (39). It is possible, though less likely, that the high IgG response observed in the RB51 shedder cow was generated independent of T cell help and driven by high and persistent levels of bacterial colonization.

Interestingly, the presence of anti-RB51 IgG in the absence of measurable peripheral CD4^+^ T cell proliferative responses has been observed previously in elk (*Cervus elaphus canadensis*) vaccinated with RB51 (40, 41). Similarly, vaccination of elk with BCG, a live attenuated *Mycobacterium bovis* strain, and the vaccine for human tuberculosis also induces IgG responses but no measurable CD4^+^ T cell response (42). We speculate that elk have a genetic predisposition for generating strong humoral responses to intravesicular organisms that typically induce strong T_H_1 responses. Since all cattle identified as carriers to date for RB51 are Jerseys, this breed or a subpopulation of this breed may have a genetic predisposition that prevents them from mounting an immune response that controls and clears the intracellular bacteria RB51. Whether these individual animals might be prone to persistent infections with other intracellular bacteria is unknown.

IgM antibodies appear first following infection or vaccination and are generally thought to provide a first line of defense against microbial challenge until class switching occurs and IgG is produced. The lack of measurable IgM in conventional cattle following the RB51 vaccination is likely due to the time points that were used to sample. With the first time point at 4 weeks post-vaccination, we speculate that we missed the IgM response, if any, elicited by vaccination. However, in the RB51 shedder cow, we observed not only a higher level of anti-RB51 IgM but also a consistently higher level of IgM following the RB51 vaccination. The sustained anti-RB51 IgM responses observed in the RB51 shedder cow may be explained by a lack of CD4^+^ T cell help, as demonstrated in this study. Without RB51-specific CD4^+^ T cells, newly activated B cells that recognize RB51 antigen in the lymph nodes, would not undergo class switching, thus leading to sustained IgM production (35). Alternatively, various studies in mice have demonstrated the existence of long-term IgM responses to various viral and bacterial pathogens. These studies suggest that such responses may be the result of long-lived IgM-secreting plasmablasts, naïve cells exposed to the chronic or persistent antigen, and/or IgM-secreting, innate-like B-1 B cells (43). At this time, we cannot determine the source of anti-RB51 IgM in the RB51 shedder cow; however, further studies analyzing the humoral response to RB51 are warranted.

These studies were limited to the analysis of the adaptive cellular and humoral responses to RB51 in the RB51 shedder animal. Yet, the data presented here cannot provide a mechanism of action to explain this phenotype. The development of adaptive immune responses, particularly the activation of T cells, is tightly linked to antigen-presenting cell (APC) activation and function. *Brucella* spp. not only infect both macrophages and dendritic cells but also alter their function to establish and maintain chronic infection through immunomodulation [reviewed in (44)]. However, further studies are needed to evaluate this. Characterization of the innate immune responses, such as those of APC and their interactions with RB51, along with genome analyses comparing possible breed-specific differences in immune genes, could help explain this response to RB51. As such, comparative transcriptomic analysis of PBMC from the RB51 shedder cow and other cattle is underway in our laboratory.

Altogether, the information presented here provides an initial assessment of the cellular and humoral immune responses in an adult cow persistently colonized by the live attenuated vaccine *B. abortus* strain RB51. To our knowledge, this analysis is the first of its kind to provide evidence that RB51 persistence is maintained by a lack of peripheral CD4^+^ T cell responses and concurrently high anti-RB51 IgG levels. Elucidating a mechanism of action would provide further understanding of the host immune response to RB51 and might facilitate insights into key components or regulation of cellular immunity in cattle. Understanding these mechanisms may also allow the development of improved interventions or vaccinations for brucellosis that would provide public health benefits.

## Data availability statement

The raw data supporting the conclusions of this article will be made available by the authors, without undue reservation.

## Ethics statement

The animal study was approved by National Animal Disease Center Institutional Animal Care and Use Committee (NADC IACUC). The study was conducted in accordance with the local legislation and institutional requirements.

## Author contributions

PB: Conceptualization, Data curation, Formal analysis, Funding acquisition, Investigation, Methodology, Validation, Writing – original draft, Writing – review & editing. HS: Data curation, Formal analysis, Methodology, Writing – review & editing. SF: Conceptualization, Formal analysis, Methodology, Writing – review & editing. KS-D: Data curation, Formal analysis, Writing – review & editing. EP: Formal analysis, Investigation, Visualization, Writing – review & editing. SO: Conceptualization, Funding acquisition, Investigation, Project administration, Resources, Supervision, Writing – review & editing.
