# Supplementary figures and images for: Characterization of the adaptive cellular and humoral immune responses to persistent colonization of Brucella abortus strain RB51 in a Jersey cow
Source: Front Vet Sci. 2024 Jul 26;11:1367498. doi: 10.3389/fvets.2024.1367498 (PMC11312097; doi:10.3389/fvets.2024.1367498)

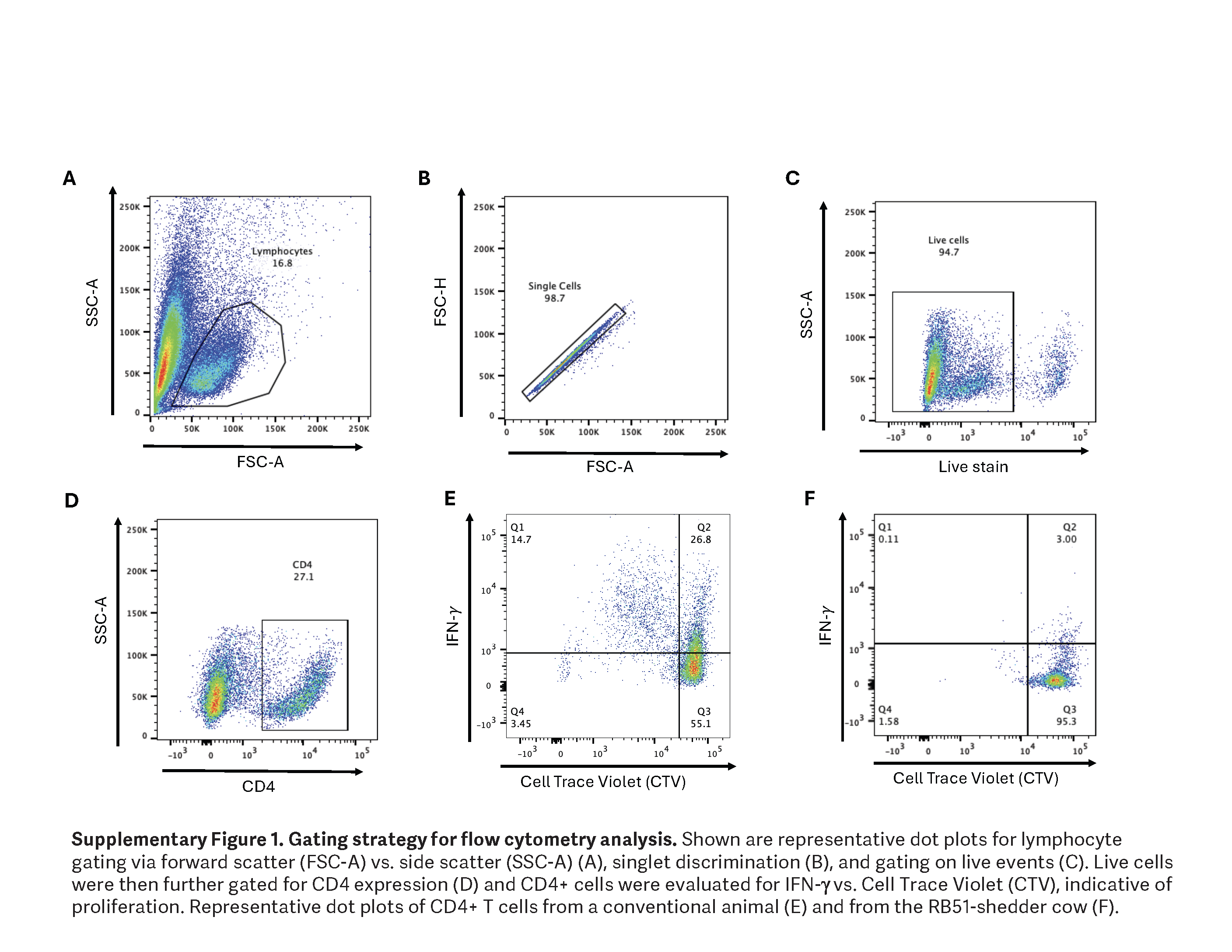

Supplement: Supplementary file 1 [file Image_1.TIFF]

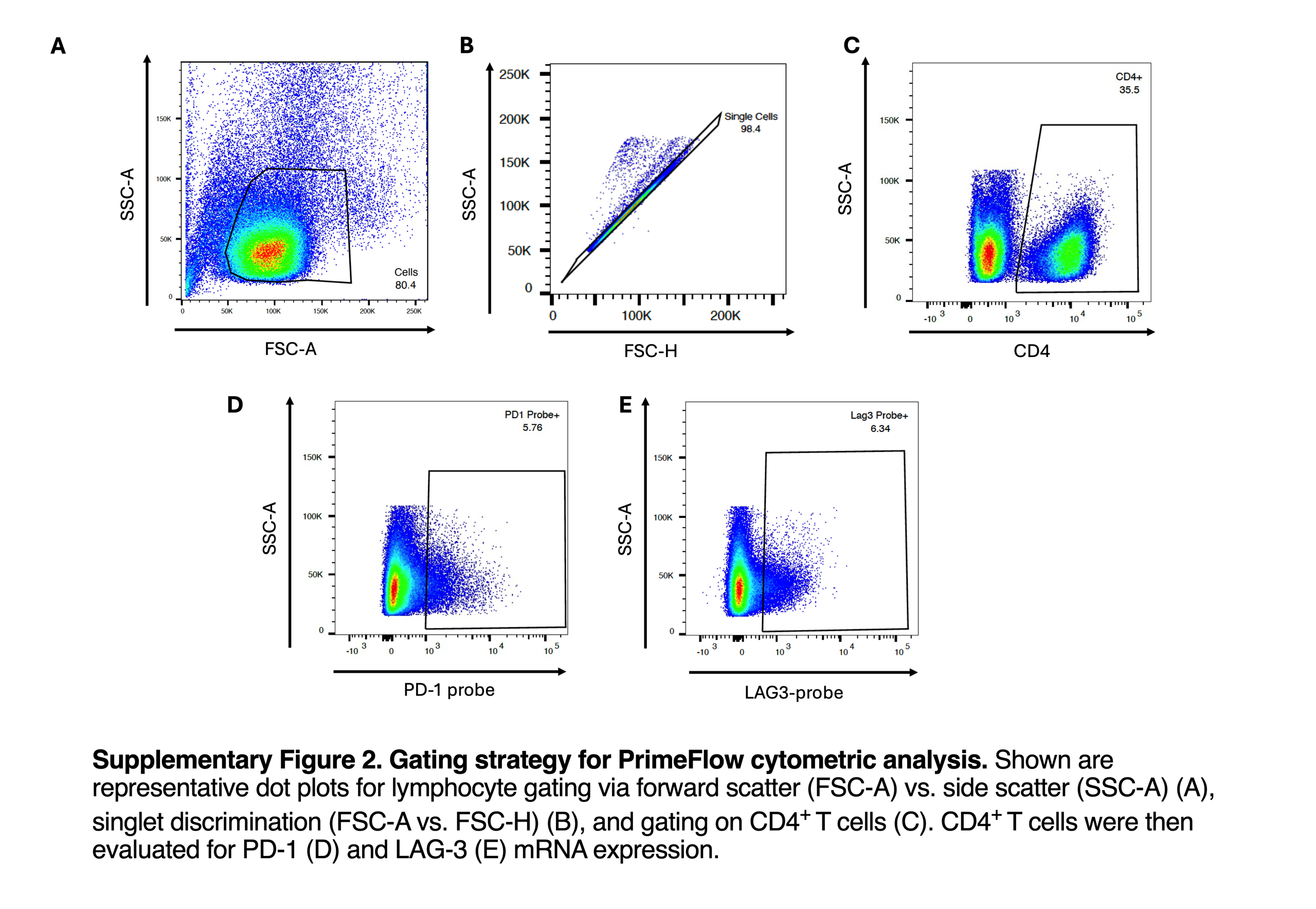

Supplement: Supplementary file 2 [file Image_2.TIFF]
